# Editorial Note: “In Weapons We Trust?” Four-culture analysis of factors associated with weapon tolerance in young males

**DOI:** 10.1371/journal.pone.0351696

**Published:** 2026-06-16

**Authors:** The PLOS One Editors

After publication of this article [1], PLOS identified concerns about the integrity of one of the article’s peer reviews. The *PLOS One* Editors investigated and concluded that the article’s publication is supported based on expert input that was not affected by the concerns.
